# Bilateral Intracardiac Microbubbles in a Patient With Giant Hiatus Hernia: A Case Report

**DOI:** 10.7759/cureus.20933

**Published:** 2022-01-04

**Authors:** Jumpei Sawa, Nozomi Nishikura, Ryuichi Ohta, Chiaki Sano

**Affiliations:** 1 Community Care, Unnan City Hospital, Unnan, JPN; 2 Community Medicine Management, Shimane University Faculty of Medicine, Izumo, JPN

**Keywords:** heart failure with reduced ejection fraction, rural hospitals, air embolism, portal vein gas, cerebral air embolism, intracardiac microbubbles, giant hiatus hernia

## Abstract

Intracardiac microbubbles may occur inadvertently during a cardiac procedure, which are typically reported in patients with central venous catheters or cardiac prosthetic valves. Here, we report a case wherein a microbubble filling in the bilateral atriums and ventricles was revealed during echocardiography despite the patient not having the aforementioned risks. An 87-year-old man with hypertension was admitted with a diagnosis of heart failure caused by a giant hiatal hernia. While awaiting hernia surgery, he started vomiting and suddenly went into a coma. A contrast-enhanced computed tomography (CT) scan of the abdomen showed a thickening of the gastric wall, intramural gas, and portal vein gas. Considering these findings, a giant esophageal hiatus hernia was suspected as the cause of the intracardiac microbubbles. In addition, an echocardiogram showed a patent foramen ovale, and the magnetic resonance imaging (MRI) of the head showed multiple cerebral infarctions bilaterally in the cerebral hemispheres. Therefore, a paradoxical air embolism was suspected to cause the coma in this patient. A giant esophageal hiatus hernia can cause portal vein gas triggered by an increased intragastric pressure (which causes vomiting). Then, the portal vein gas flows into the right heart via the sinusoids. Cerebral air embolism can also develop via a shunt, such as a patent foramen ovale, and trigger a foreign body reaction via inflammation and cause coma. When microbubbles are observed in the heart on an echocardiogram, it is necessary to seek the place of entry because it can be a lethal sign due to complications that could follow, such as a cerebral air embolism or pulmonary air embolism.

## Introduction

Iatrogenic detection of microbubbles is common in patients with transcutaneous lines, certain medical devices, or cardiac prosthetic valves [1]. They have been associated with intestinal ischemia and colon cancer as well and may be generated by absorbed intestinal gas that reaches the heart through the portal system and systemic vein shunts [1-3]. The routes for the air in the heart can vary based on abnormal anatomy of shunts such as a patent foramen ovale, aorta-esophageal, and vena cava-duodenal fistula [1-3].

Here, we report a case of an 87-year-old patient with hypertension, in whom the microbubbles occurred in the right heart system unexpectedly. The microbubbles circulated throughout the body via a right-to-left shunt. The echocardiography showed the microbubble filling up the cardiac cavities bilaterally.

## Case presentation

An 87-year-old man presented to the hospital with a complaint of anorexia for two weeks. He had been well until two weeks prior to the consultation, when he had a coffee-ground emesis. On his first visit to our hospital, he was diagnosed with gastroesophageal reflux disease and macrocytic anemia, and lansoprazole and folic acid were administered. Although his vomiting improved, he developed anorexia and his family doctor referred him to our hospital. In the past two weeks, he gained 5 kg in weight and developed edema in both legs. He had no complaints of dyspnea, chest pain, fever, or chills. His medical history included hypertension and gastroesophageal reflux disease, and no history of smoking. His medications at the time of admission included ferrous citrate, folic acid, lansoprazole, mosapride, and bifidobacteria. There were no abnormal vital signs except for tachypnea at 24 beats per minute.

On physical examination, the jugular vein was distended, and the chest auscultation revealed bilateral crackles. There was bilateral pitting edema in the lower extremities. Blood tests revealed a decreased hemoglobin of 8.2 g/dL, albumin of 2.5 g/dL, and elevated brain natriuretic peptide (BNP) level of 448 pg/mL. Other laboratory test results are shown in Table 1.

**Table 1:** Laboratory data on admission and on day 17 of hospitalization. PAO_2_, partial pressure of oxygen; PACO_2_, partial pressure of carbon dioxide

Marker	Day 1	Day 17	Range
White blood cells	4.5	5.3	3.5–9.1 × 10^3^/μL
Neutrophils	78.7	76.4	44.0–72.0%
Lymphocytes	7.7	17.1	18.0–59.0%
Monocytes	12	5.9	0.0–12.0%
Eosinophils	0.7	0.4	0.0–10.0%
Basophils	0.5	0.2	0.0–3.0%
Red blood cells	2.37	2.74	3.76–5.50 × 10^6^/μL
Reticulocytes (%)	4.2		0.2–2.0%
Hemoglobin	8.2	9.1	11.3–15.2 g/dL
Hematocrit	25.4	28.1	33.4–44.9%
Mean corpuscular volume	107.2	102.4	79.0–100.0 fL
Platelets	19.8	16.0	13.0–36.9 × 10^4^/μL
Total protein	4.8	5	6.5–8.3 g/dL
Albumin	2.5	2.4	3.8–5.3 g/dL
Total bilirubin	0.3	0.3	0.2–1.2 mg/dL
Aspartate aminotransferase	23	22	8–38 IU/L
Alanine aminotransferase	16	21	4–43 IU/L
Alkaline phosphatase	88	71	106–322 U/L
Γ-glutamyl transpeptidase	35	26	<48 IU/L
Blood urea nitrogen	17.3	29.1	8–20 mg/dL
Creatinine	0.71	0.57	0.40–1.10 mg/dL
Serum sodium	134	131	135–150 mEq/L
Serum potassium	4.8	3.2	3.5–5.3 mEq/L
Serum chloride	101	92	98–110 mEq/L
Serum calcium	7.8	8	3.5–10 mg/dL
C-reactive protein	0.61		<0.30 mg/dL
Ferritin	39.5		14.4–303.7 ng/mL
Brain natriuretic protein	448		<40 pg/mL
Troponin I		0.018	<0.02 ng/mL
D-dimer		4.1	<1 μg/mL
pH		7.458	
PaO_2_		56.4	80-100 mmHg
PaCO_2_		41.6	35-45 mmHg
Serum bicarbonate		29.5	22-26 mmol/L
Lactate		3.1	0.26-1.39 mmol/L

**Figure 1:**
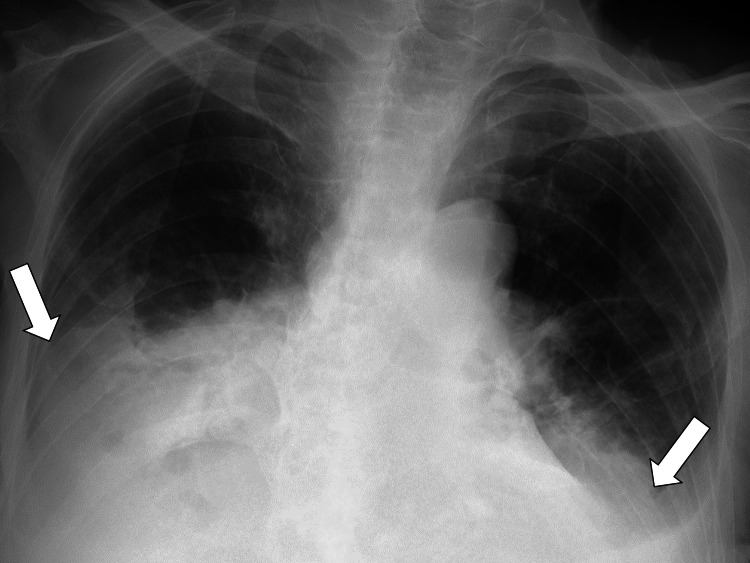
Chest X-ray at admission The bilateral costophrenic angles were dull.

An electrocardiogram (ECG) revealed a sinus rhythm and complete right bundle branch block. Chest radiography showed a bilateral costophrenic angle blunt (Figure 1).

The patient was admitted to the hospital and treated for acute heart failure with intravenous furosemide at 40 mg/day. The patient responded to treatment over several days, with weight loss and an improvement in both leg edema and tachypnea; however, his anorexia did not improve.

**Figure 2:**
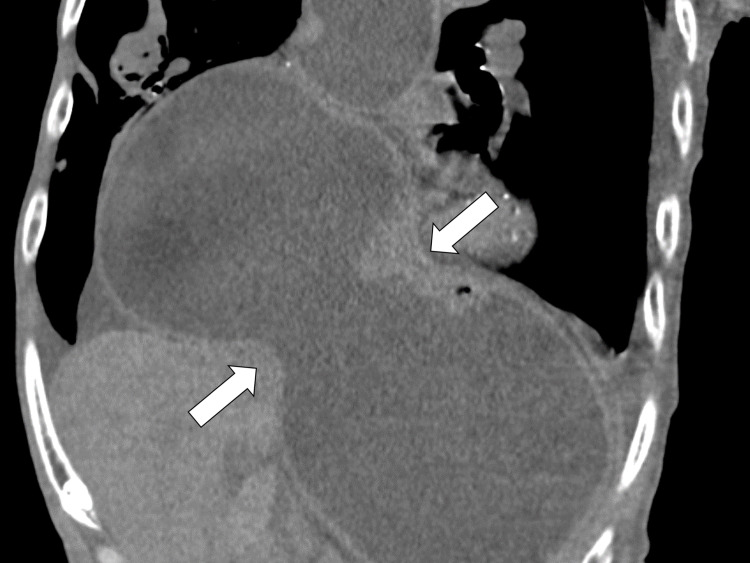
Abdominal CT scan on the fifth day The CT revealed a giant esophageal hiatal hernia. CT, computed tomography

On the fifth day of hospitalization, a computed tomography (CT) scan was performed to investigate the cause of his anorexia, which revealed a giant esophageal hiatus hernia (Figure 2).

**Figure 3:**
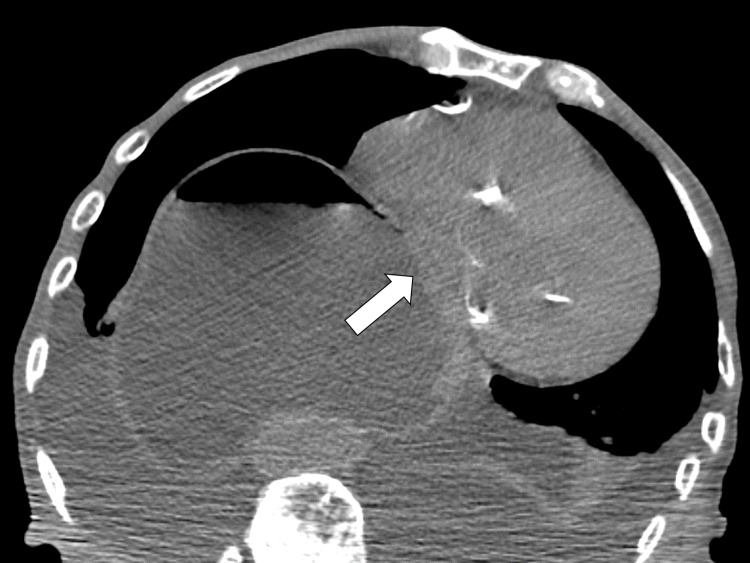
Abdominal CT scan on the fifth day The CT revealed a giant esophageal hiatal hernia compressing the heart anteriorly. CT, computed tomography

The giant esophageal hiatus hernia compressed the heart anteriorly (Figure 3).

There were no other abnormalities in the abdominal cavity that could cause anorexia or vomiting. We concluded that his anorexia was caused by a giant hiatus hernia, and the compression of the hernia in the atrium caused heart failure. Surgery was planned for the repair of a giant esophageal hiatal hernia one week later. However, he could not progress to the surgery.

**Figure 4:**
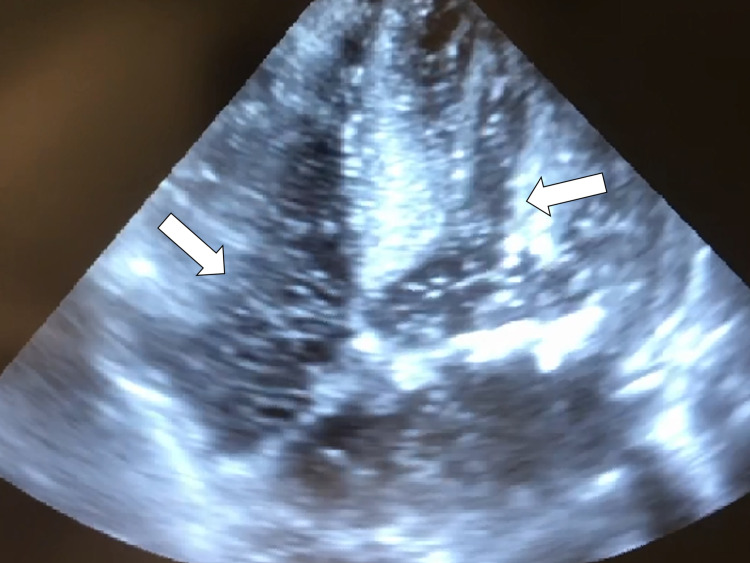
Echocardiography on the 17th day The echocardiography revealed microbubbles in bilateral cardiac cavities.

On the 17th day, the patient suddenly went into a coma after vomiting. Regarding his consciousness, his Glasgow Coma Scale (GCS) score was 6. His respiratory rate was 24 breaths per minute, pulse was 85 beats per minute, blood pressure was 110/80 mmHg, and SpO_2_ was 83%, while breathing oxygen through an oxygen mask at a rate of 4 L/minute. On physical examination, the pupils were 3 mm in diameter, equal, and reacted to light, and the jugular vein was not distended. The lungs were clear on auscultation, and the cardiac auscultation revealed no murmur. Blood gas analysis showed a lactate level of 3.1 mmol/L; other laboratory data are shown in Table 1. Only transthoracic echocardiography was performed to investigate the hypoxemia and elevated lactate levels, which revealed microbubbles in the bilateral cardiac atriums and ventricles with a tiny foramen ovale (Figure 4).

**Figure 5:**
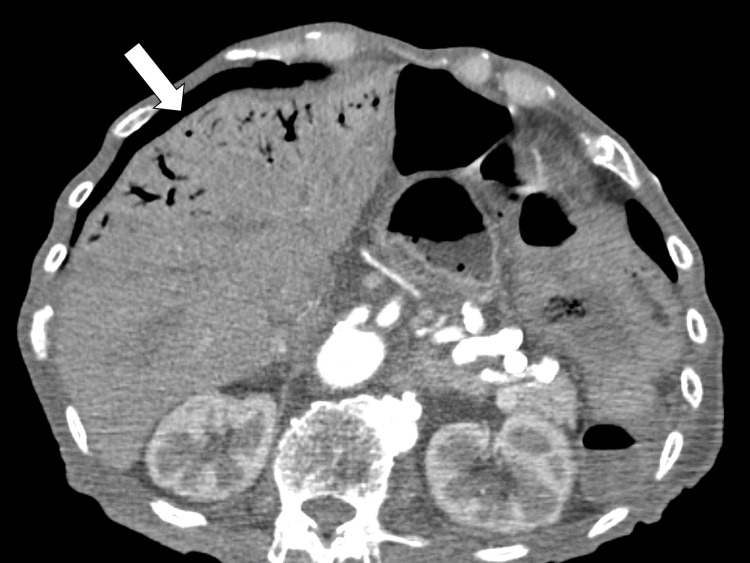
Contrast CT scan on the 17th day The CT revealed portal vein gas in the liver. CT, computed tomography

**Figure 6:**
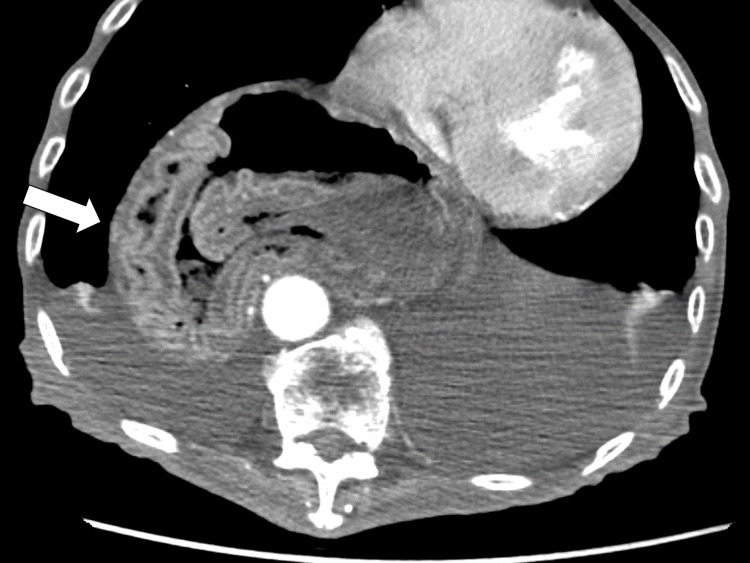
Contrast CT scan on the 17th day The CT revealed gastric wall thickening. CT, computed tomography

The contrast-enhanced CT showed portal vein gas (Figure 5) in the liver gastric wall thickening (Figure 6).

There were no contrast defects in the main pulmonary artery trunk or the intestinal wall. A head MRI on the 18th day showed multiple cerebral infarctions bilaterally in the cerebral hemispheres, and transthoracic echocardiography showed a right-to-left shunt in the atrium. We could not perform transesophageal echocardiography and measure the size of the foramen because of the emergency. We suspected that the microbubbles entered the venous system via the hiatus hernia and passed to the arterial system through the patent foramen ovale and subsequently caused a cerebral air embolism. The patient’s family refused further investigations and treatment; he was therefore treated palliatively. He died on the 20th day after admission.

## Discussion

In this case, the patient with a giant esophageal hiatus hernia went into a comatose state, and a transthoracic echocardiography revealed microbubbles. We inferred that the esophageal hiatus hernia was the microbubbles’ point of entry. The microbubbles then entered the systemic circulation through a patent foramen ovale, ultimately causing a cerebral air embolism.

When intracardiac microbubbles are found in patients without medical devices, it is important to identify the entry point [3]. In our case, the patient seemed to have a giant esophageal hiatal hernia. In addition, if microbubbles were found in both sides of the heart, a right-to-left shunt should be investigated. In this case, the patent foramen ovale caused a right-to-left shunt. Here, we present previous literature to support our theory about the microbubbles’ journey from the esophageal hiatus hernia to the brain.

The first part of this route is the route to the portal vein. As the mechanism of portal vein gas is not entirely understood, there are various possible explanations for this finding. Theories include (1) disruption of the intestinal lumen with passage of gas into the mesenteric venous system from gas-forming organisms within the intestinal mucosa, (2) the presence of gas-producing organisms in an abscess or portomesenteric pylephlebitis, and (3) migration of gas through the mural capillaries into the portal venous system due to high gastrointestinal luminal pressure. Most cases of portal vein gas are caused by mesenteric vascular occlusion and subsequent intestinal necrosis. In these cases, the first theory above is a likely mechanism. Colonic diverticulitis with abscess formation has been reported to cause portal vein gas with the second theory above as a possible mechanism. The stomach rarely causes portal vein gas [4,5], in which the third theory above is assumed to be the underlying mechanism. Gastric dilatation and gastric emphysema have been reported as the major causes of increased internal pressure [6]. An esophageal hiatus hernia has also been reported to cause portal vein gas as a result of gastric dilatation due to strangulation [6]. In this case, vomiting might have caused increased gastric pressure at the site of the hernia, and the gas produced from gastric dilatation might have migrated through the gastric wall into the portal vein.

The next part of this route is from the portal vein to the systemic vein. Portal vein gas can pass to the systemic vein via two routes: through the liver and through a portosystemic shunt [7-9]. In this case, the flow of microbubbles from the hepatic vein to the inferior vena cava was observed, suggesting that the bubbles passed through the sinusoids to the inferior vena cava.

Microbubbles were observed bilaterally in the cardiac cavities. This suggests that the bubbles from the right ventricular system entered the left ventricular system. Usually, bubbles in the right ventricle are trapped in the pulmonary capillaries after passing through the pulmonary artery and would not flow into the left ventricle. There are several mechanisms underlying this, of which one is a right-to-left shunt through the heart or lungs, such as a patent foramen ovale. For example, the Valsalva maneuver can transiently increase right atrial pressure, creating a right-to-left shunt and transferring the potential embolic source into the systemic circulation. Similarly, increasing pulmonary artery pressure caused by a pulmonary embolism also increases right-to-left shunt flow [10,11]. In this case, transthoracic echocardiography showed blood flow between the right and left atria, suggesting a patent foramen ovale. Vomiting (observed in this case before the onset of the coma) might have caused an increased intrathoracic pressure and accelerated shunt flow. The other mechanism involves the volume of the microbubbles. When it is a large volume of microbubbles, a passage of air emboli from the venous to the arterial circulation can occur [1]. In this case, a large volume of air bubbles was observed in both heart chambers continuously, suggesting the possibility of air inflow by this mechanism.

Finally, the microbubbles that entered the systemic circulation had reached the brain through the aorta, causing a cerebral air embolism. More than 5% of cases of cerebral infarction are speculated as caused by a patent foramen ovale and called as a paradoxical embolism [12]. In cerebral infarction, the clinical presentation is determined by the areas of the brain that are affected, and complete disorientation is rare [12]. However, cerebral infarction due to an air embolism is known to cause coma [11]. This may be related to the pathophysiology of the microbubble embolization [11]. In addition to ischemia (due to vascular occlusion), an inflammatory response occurs because the body reacts with the bubble as a foreign substance [12]. Both processes result in vasogenic edema, and neuronal injury extends beyond the site of obstruction [12]. In this case, the degree of disorientation was much more significant than the imaging findings, which may be due to the characteristics of such air embolization.

The learning points from this case report are that if right ventricular air is present on echocardiography, the point of entry should be found, which suggests gastrointestinal ischemia or increased internal pressure; giant esophageal hiatus hernia can cause portal vein gas and intracardiac microbubbles triggered by increased intragastric pressure due to vomiting; if echocardiography shows bubbles in both heart cavities, an intracardiac shunt (such as an open foramen ovale) should be sought; and if echocardiography shows intracardiac microbubbles in patients a comatose condition, a cerebral air embolism should be included in the differential diagnosis.

## Conclusions

Microbubbles entering the venous system can travel to the systemic vein, which can then enter the systemic circulation via a right-to-left shunt and cause a paradoxical cerebral air embolism. Since the underlying cause is lethal and could lead to life-threatening complications, it is important to investigate the entry point whenever intracardiac microbubbles are observed. A giant esophageal hiatus hernia is a rare but possible cause. Surgical treatment of a giant esophageal hiatus hernia may prevent the development of microbubbles.
